# Photoreactivity of Bis-retinoid A2E Complexed with a Model Protein in Selected Model Systems

**DOI:** 10.1007/s12013-020-00942-1

**Published:** 2020-09-12

**Authors:** Justyna Furso, Andrzej Zadlo, Grzegorz Szewczyk, Tadeusz J. Sarna

**Affiliations:** Department of Biophysics, Faculty of Biochemistry, Biophysics and Biotechnology, Jagiellonian University, 30-387 Krakow, Poland

**Keywords:** A2E, Photochemical reactivity, Protein oxidation, Reactive oxygen species, Singlet oxygen, EPR-Spin trapping

## Abstract

The bis-retinoid N-retinyl-N-retinylidene ethanolamine (A2E) is formed as a byproduct of visual cycle in retinal pigment epithelium (RPE). It contributes to golden-yellow fluorescence of the age pigment lipofuscin, which accumulates in RPE. Lipofuscin can generate a variety of reactive oxygen species (ROS) upon blue-light excitation. Although in model systems photoreactivity of A2E has been determined to be low, this bis-retinoid exhibited significant phototoxicity in RPE cells in vitro. Although the mechanism of A2E-mediated phototoxicity remains mostly unknown, we hypothesize that formation of A2E-adducts with different biomolecules may play an important role. In this study, we investigated the photochemical reactivity of A2E and its complex with bovine serum albumin (BSA) using UV–Vis absorption and emission spectroscopy, EPR-spin trapping, EPR-oximetry, time-resolved singlet oxygen phosphorescence, and the fluorogenic CBA probe. Our data show that A2E after complexation with this model protein photogenerated an increased level of ROS, particularly singlet oxygen. We also demonstrated the ability of A2E to oxidize BSA upon excitation with blue light in aqueous model systems. The data suggest that pyridinium bis-retinoid could oxidatively modify cellular proteins under physiological conditions.

## Introduction

**Scheme 1:**
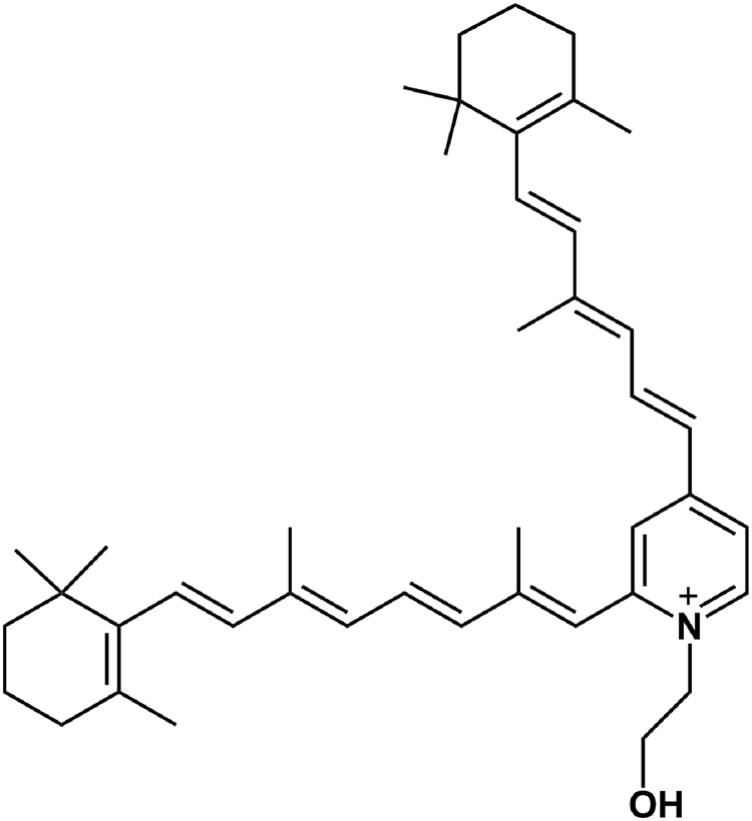
The structure of A2E

It has been postulated that the amorphous pigment lipofuscin, which accumulates with age in retinal pigment epithelium (RPE), mediates photochemical reactions, which can contribute to oxidative stress in the outer retina. It has been documented that lipofuscin, can generate, upon excitation with blue light, reactive oxygen species (ROS) [1, 2], participate in lipid and protein oxidation, inactivate lysosomal and antioxidant enzymes [3–6] and inhibit phagocytic activity of RPE cells in vitro [7]. Chronic oxidative stress in RPE cells may contribute to the development of variety of retinal degenerative diseases [8, 9] including age-related macular degeneration, in which the role of lipofuscin has been generally considered [10–13]. Lipofuscin is a composite granule containing lipids, proteins and several pigments which absorb blue light [14]. One of such chromophores is N-retinylidene-N-retinylethanolamine, called A2E (Scheme 1), formed mostly nonenzymatically from the metabolites of the visual pigment and lipid components of photoreceptor outer segment membranes [15]. This pyridinium bis-retinoid has been identified as an important fluorescent chromophore of the RPE age pigment [16–18]. Biosynthesis of A2E takes place in RPE, where lipofuscin granules accumulate with senescence. The concentration of A2E in RPE may reach 20 µM in advanced age [19]. A2E is composed of two hydrophobic polyene side-chains and a polar positively charged pyridinium moiety. Such structure results in detergent-like nature of the compound. As a result, this bis-retinoid may act destructively in RPE cells even without light exposure by disrupting organelle membranes, such as lysosomal membranes. It was also demonstrated that incorporation of A2E into model membranes accelerated cholesterol displacement from lipid bilayers. If such a phenomenon occurs in RPE cells, it would result in accumulation of free and esterified cholesterol in the cells [20]. The disturbed degradative functions of RPE lysosomes mediated by A2E was explained by inhibition of the ATP-driven proton pump in organelle membranes [21, 22].

A2E is also known to be photoreactive. It absorbs light in the UV and visible region and its absorption spectrum in organic solvents features two distinct maxima at 336 and 439 nm [23]. It has been postulated that A2E is at least partially responsible for photoreactivity and phototoxicity of lipofuscin. Interestingly, although in simple model systems the photochemical reactivity of A2E was shown to be low [24], it exhibited significant phototoxicity in RPE cells in vitro [25, 26].

The ability of A2E to photoinduce peroxidation of proteins has not been systematically investigated. However, such a process may have significant implications, especially in the case of RPE proteins responsible for the cell architecture. We believe that oxidative modifications of cytoskeletal proteins may have adverse effect on both morphology and nanomechanical properties of RPE cells [27, 28]. Recently, Wiktor et al. [29]. have demonstrated that sublethal or weakly lethal photic stress, mediated by human RPE lipofuscin granules, affected nanomechanical properties of ARPE-19 cells. It turned out that the photogenerated oxidative stress brought about oxidation of cellular proteins, disrupted the cell cytoskeleton, and modified the cell elasticity. It is important to emphasize that the role of A2E in these phenomena remains unknown. The accumulating in the retinal tissue A2E may form complexes with proteins and other cellular constituents that could modify this bis-retinoid photoreactivity and phototoxicity. Therefore, it is relevant to examine how complexation with a model protein changes the ability of A2E to photogenerate ROS.

In this study, we investigated the effect of bovine serum albumin (BSA) on photochemical reactivity of A2E using EPR-spin trapping, EPR-oximetry, and singlet oxygen detection. We also analyzed the capability of A2E to oxidize BSA using the sensitive coumarin boronic acid (CBA) probe and fluorescence measurements.

## Materials and Methods

### Chemicals

The following chemicals were obtained from Sigma-Aldrich (Steinheim, Germany or St. Louis, MO, USA): all-*trans* retinal, ethanolamine, trifluoroacetic acid (TFA), methanol, ethanol, catalase, superoxide dismutase (SOD), sodium azide, 1,4-diazabicyclo[2.2.2]octane (DABCO), 4-(1,1,3,3-tetramethylbutyl)phenyl-polyethylene glycol (TX-100), chelating resins Chelex 100, diethylenetriaminepentaacetic acid (DTPA), Tetra(4-N,N,N-trimetylanilinium) porphine (TMAP), and deuterium oxide. BSA was purchased from BioShop Canada (Burlington, Canada). 4-Protio-3-carbamoyl-2,2,5,5-tetraperdeuteromethyl-3-pyrroline-1-yloxy (mHCTPO) was a gift from Professor H. J. Halpern (University of Chicago, Chicago, IL). *n*-5,5-Dimethyl-1-Pyrroline-N-Oxide (DMPO) was obtained from Dojindo Laboratories (Kumamoto, Japan). Dodecyl-β-D-maltopyranoside (DDM) was purchased from Anatrace Products LLC (Maumee, OH, USA). Disodium hydrogen phosphate dodecahydrate (Na_2_HPO_4_ × 12H_2_O), potassium dihydrogen phosphate (KH_2_PO_4_), hydrochloric acid (HCl), acetic acid, potassium chloride (KCl), sodium chloride (NaCl), acetonitrile and dimethyl sulfoxide (DMSO) were purchased from Polskie Odczynniki Chemiczne (Gliwice, Poland). CBA indicator was a gift from Professor A. Sikora (Institute of Applied Radiation Chemistry, Technical University of Lodz, Poland). All chemicals were of highest purity and used as supplied. All working solutions were prepared with double distilled water and stored at 4 °C or at room temperature.

### Synthesis and Purification of A2E

A2E was synthesized as previously reported by Parish et al. [30]. Two equivalents of all-*trans* retinal, one equivalent of ethanolamine and one equivalent of acetic acid in ethanol where stirred at room temperature for 48 h in the dark. A2E was pre-purified by silica gel chromatography and then purified by HPLC (Shimadzu, Japan) on the semipreparative Cosmosil 5C18 RP column, 250 × 10 mm (Beckman Coulter, USA) with the following gradients of acetonitrile/water (0.1% TFA): 90–100% for 0–10 min, 100% acetonitrile (0.1% TFA) for 10–35 min (flow rate 4 mL/min). Elution of the A2E was monitored at 430 nm and its retention time was 9 min. Identification of A2E was determined by UV–Vis spectrophotometry (Hitachi U-2900, Japan). Stock solution of A2E was prepared in acetonitrile or methanol and kept in −80 °C.

### Preparation of A2E-BSA Complexes

BSA stock solution was prepared in H_2_O or D_2_O and kept prior to use in 4 °C. A2E-BSA complex was prepared by adding a small volume of concentrated A2E in acetonitrile directly to a solution of BSA in water, mixed vigorously and kept on ice. In some experiments A2E and BSA were suspended in micellar model system containing 1% of TX-100 or 1% of DDM in H_2_O or D_2_O. Final concertation of A2E and BSA in a non-micellar and micellar model system, depending on the exact experiment, was 10 µM or 50 µM and 20 µM or 100 µM, respectively.

### Oxygen Consumption Measurements

Rates of photoconsumption of oxygen were obtained by measuring kinetics of oxygen concentration changes in irradiated samples by EPR oximetry, according to method described elsewhere [31, 32]. Suspension of A2E (50 µM) or/and BSA (100 µM) in water/micellar system containing 100 µM mHCTPO used as an oxygen-sensitive spin probe, was placed in a standard EPR flat quartz cell and positioned in a resonant cavity equipped with an optical window. Oxygen consumption was examined during in situ irradiation of samples with blue light (30 mW/cm^2^) derived from a 300 W high pressure compact arc xenon lamp (Cermax, PE300CE–13FM/Module300W, Perkin-Elmer) equipped with a water filter, heat reflecting hot mirror, and dichroic filter transmitting light at a range of 402–508 nm. To examine a possible role of superoxide anion, in the photo-induced oxygen uptake in samples containing A2E/BSA in D_2_O, EPR oximetry was also carried out after addition of 500 U/mL of SOD. Such samples were irradiated with 10 mW/cm^2^ blue light. The EPR measurements were performed at ambient temperature using a Bruker EMX–AA EPR spectrometer (Bruker BioSpin, Rheinstetten, Germany) operating at 9.5 GHz with 100-kHz field modulation. EPR spectra were registered at following instrument settings: microwave power 1 mW, modulation amplitude 0.006 mT, scan width 0.5 mT, and scan time 12 s. Rates of oxygen uptake were determined by linear fitting to initial points of the oxygen depletion plot.

### Singlet Oxygen Measurements

Time-resolved phosphorescence of ^1^O_2_ was detected at 1270 nm in 1% DDM-D_2_O system and D_2_O. Concentrations were adjusted to obtain OD = 0.17 at 422 nm for both the A2E sample and reference photosensitizer. Samples were measured in 10 mm fluorescence cuvette (QA-1000, Helma, Germany) and excited with 422 nm pulses of 3.6 ns duration emitted by Nd:YAG laser (NT242, Expla, Lithuania) operating at 1 kHz repetition rate. To adjust photoexcitation energy, the laser beam was attenuated with three pieces of a wire mesh (light transmission of each piece ~ 35%). The exciting laser beam was perpendicular to the detection path, and the luminescence signals were recorded in a photon counting mode using a thermoelectric-cooled NIR PMT unit (Model H1033045, Hamamatsu, Japan) equipped with a 1100-nm cutoff filter and additional selected narrow-band filter (NB series, NDC Infrared Engineering LTD, UK). The data acquisition was performed using a computer-mounted PCI-board multichannel scaler (NanoHarp 250, PicoQuant GmbH, Germany). Data were collected for 15 s. The data analysis was performed by a custom-written software and included first-order luminescence decay fitting by the Levenberg–Marquardt algorithm. Determination of singlet oxygen quantum yield (*ϕ*_Δ_) generated by A2E in 1% DDM-D_2_O was carried out relative to TMAP (Tetra(4-N,N,N-trimetylanilinium)porphine) as the reference sensitizer, with a *ϕ*_Δ_ = 0.73 [33].

### EPR-spin Trapping

For detection of superoxide anion, 5,5-dimethyl-l-pyrroline-N-oxide (DMPO; 100 mM) was used as a spin trap. Photo-induced generation of superoxide was monitored in A2E or A2E/BSA samples dissolved in 80% DMSO. DMPO has a low efficacy for trapping superoxide radicals in aqueous media and the DMPO-OOH adduct is quite unstable [34]. However, since superoxide has nucleophilic character in aprotic solvents [35], their application facilitates its spin trapping. Therefore, the superoxide spin trapping experiments were carried out using high concentration of DMPO and the highest possible DMSO content (80%). The other 20% of the sample solvent was water used for preparation of BSA and DMPO stock solutions. Samples with and without 500 U/ml of SOD in quartz EPR flat cells were place in resonant cavity and irradiated with blue light. To examine possible effects of singlet oxygen, spin trapping experiments were also carried out after the addition of 10 mM sodium azide or 10 mM DABCO, efficient quenchers of this ROS [36]. The photo-induced formation of free radicals was inferred from the observed accumulation of characteristic spin adducts, and their identification was confirmed by simulation of the experimental spectra using WinSIM software. Irradiating light, with similar parameters, was derived from the same light source as that used in oxygen photoconsumption experiments. The EPR samples were run using a microwave power 10 mW, modulation amplitude 0.05 mT, sweep width 12 mT, and sweep time 42 s.

### Determination of Protein Oxidation by CBA Assay

Photooxidation of albumin in micellar and non-micellar model systems mediated by A2E was determined by monitoring the conversion of the nonfluorescent CBA into fluorescent 7-hydroxycoumarin (COH) as described elsewhere [37, 38]. Immediately after blue-light irradiation (440 nm; 60 mW/cm^2^), control samples with BSA (100 µM) and samples containing A2E (50 µM), were incubated with catalase (250 U/ml) for 5 min then frozen in liquid nitrogen. Some samples in D_2_O-micellar systems were blue light-irradiated with or without 5 mM sodium azide. All samples were transferred at the same time into black 96-well plates and incubated with phosphate buffer (PB) (50 mM, pH 7.4), DTPA (100 µM), CBA (200 µM), and catalase (250 U/ml). Fluorescence emission of the formed COH in irradiated samples was measured at 465 nm after excitation at 360 nm, using the ClarioStar plate reader (BMG Labtech, USA). Due to low reactivity of the probe with protein hydroperoxides, the fluorescence signal was recorded at 10 min intervals for 20 h. Fluorescence was plotted against time as shown in Fig. 8. The extent of protein oxidation was given as a concentration of the albumin hydroperoxides calculated from the crossing points of lines fitted to the initial increase of the fluorescent intensity and the last points of the increase of fluorescent intensity of the same sample and normalized to the hydrogen peroxide calibration curve. Samples for the calibration were prepared in similar manner as examined samples except the catalase addition. The experiments were repeated minimum two times.

### Fluorescence Measurements

Emission spectra of A2E and BSA in PB were recorded in 10 mm fluorescence quartz cuvette (QA-1000, Helma, Germany) containing a magnetic stirrer and sealed with a rubber septum using LS55 spectrofluorimeter (PerkinElmer, Inc., USA). Prior the measurement, the sample was stirred under argon (gas was streaming on the liquid surface) for 30 min. Emission spectra for BSA and A2E were recorded at excitation wavelength 278 nm (excitation slit 10 nm/emission slit 3 nm) and 450 nm (excitation/emission slit 10 nm), respectively.

The interaction of A2E and BSA was examined in the samples prepared in PB (H_2_O) or PB (H_2_O) with 0.25% DDM. A total of 5 µM BSA was titrated with 0.575 µM A2E portions of stock solution in acetonitrile for a final A2E concentration of ~20 µM. In this range of the concentrations used, optical density (OD) of the samples was maintained at 0.2–0.4. After argon saturation the fluorescence spectra of the BSA sample with increasing concertation of A2E was recorded. The binding of A2E to BSA was investigated using the exponential decay fitted to the intensity of the BSA emission at fluorescence maximum plotted against the concertation of titrated A2E. Fitting the data to the nonlinear regression allowed us to obtain a dissociation constant (*K*_d_) of the binding pair. Quenching of the BSA fluorescence was observed at 350 and 335 nm for BSA in the solution and in the micellar system, respectively. In order to verify the negligible effect of the acetonitrile on the BSA emission, fluorescence of the protein was also measured by repeating the titration without the ligand.

Intrinsic fluorescence of BSA was measured before and after photolysis, using samples prepared in PB (D_2_O). In total, 50 µM A2E and 100 µM BSA were irradiated with 440 nm (60 mW/cm^2^) for selected time intervals. Samples were gently stirred and temperature was maintained at 4 °C during the photolysis. Before and after irradiation, 300 µL of the sample was diluted to obtain 10 µM A2E and 20 µM BSA, and the fluorescence spectra were recorded after excitation at 278, 325, and 365 nm, according to absorption maxima of tryptophan, N′-formylkynurenine, and kynurenine, respectively [39].

### Circular Dichroism (CD)

CD spectra of the samples containing 100 µM BSA or 100 µM BSA and 50 µM A2E in PB-D_2_O were recorded before and after irradiation with 440 nm light (60 mW/cm^2^) for selected time intervals. CD measurements were performed in the Department of Physical Biochemistry (Faculty of Biochemistry, Biophysics and Biotechnology, Jagiellonian University), using a Jasco J-710 spectropolarimeter (Jasco Analytical Instruments, USA). The spectra were collected in a range of 180–250 nm with a 1 nm data pitch, a 50 nm min^−1^ scanning speed, a 2 s response time and a 2 nm bandwidth and averaged over three acquisitions. Protein samples were measured in a cuvette with a 0.01 mm light path. Buffer used for sample preparation was used as a blank. The contents of secondary structure were calculated based on experimental data and Prot4 reference base using Jasco Secondary Structure Estimation (JSSE) software.

### Photodegradation of A2E

Photodegradation of air-equilibrated or saturated with argon for 2.5 h samples, containing 50 µM A2E in 1% DDM in PB-D_2_O or in the presence of 100 µM BSA in PB-D_2_O, was induced by irradiation with blue light, employing 440 nm LED (60 mW/cm^2^), and monitored by UV–Vis spectroscopy (Hitachi U-2900, Japan). During the photolysis samples were gently stirred. Samples which were blue-light irradiated under air were diluted five times for the absorption measurements.

## Results and Discussion

**Fig. 1:**
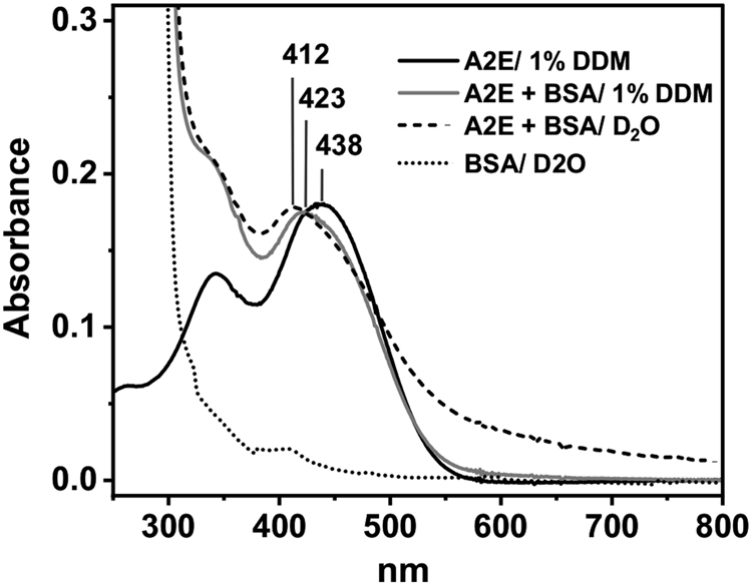
UV–Vis spectra of A2E in D2O/1% DDM (black line), with BSA in D_2_O-micellar model system (gray line), A2E-BSA complex in D_2_O (dashed line) and BSA in D_2_O (dotted line)

A2E is not soluble in water, therefore 1% DDM, forming micellar system, was used. Absorption spectrum of DDM-solubilized A2E is shown in Fig. 1. The more pronounced peak is localized at 438 nm. Addition to such sample 0.1 mM BSA, causes broadening and a small hypsochromic shift of the A2E absorption, which is particularly evident for the longer wavelength peak. Similar spectrum is also observed for A2E in the presence of 0.1 mM BSA, without the detergent; however, the sample may be partially aggregated, which is suggested by the apparent absorption in the region above 550 nm probably due to light scattering. The absorption changes induced by the addition of BSA suggest that A2E forms noncovalent complexes with BSA.

### Blue Light-induced Oxygen Consumption

**Fig. 2:**
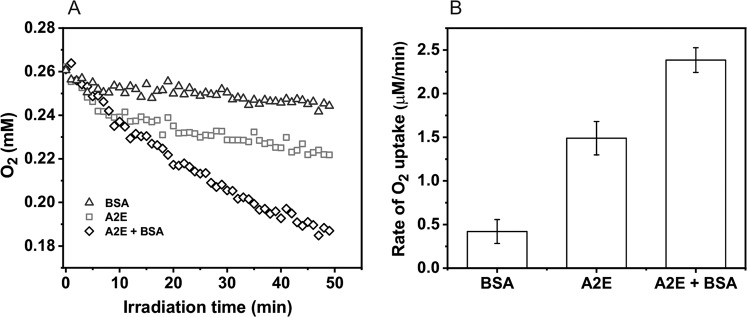
Blue light-induced oxygen uptake in the micellar model system containing 50 μM A2E, 100 μM BSA, or a suspension of both in 1% TX-100 (**a**) and determined rates of oxygen photoconsumption (**b**)

**Fig. 3:**
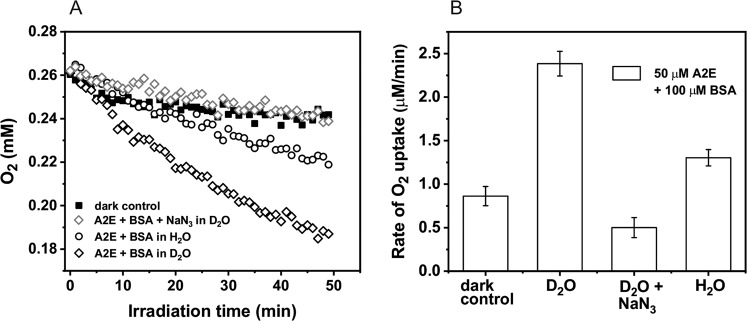
Dark and blue light-induced oxygen uptake in the micellar model system containing 50 μM A2E and 100 μM BSA in H_2_O or D_2_O (**a**) and determined rates of oxygen photoconsumption (**b**)

**Fig. 4:**
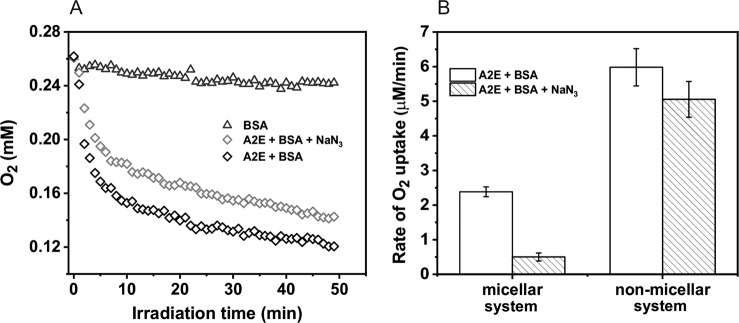
Blue light-induced oxygen uptake of A2E-BSA complex in D_2_O (**a**) and the comparison of oxygen consumption rates of A2E and BSA in D_2_O-micellar system with the A2E and BSA in D_2_O-non-micellar system (**b**)

The A2E photoreactivity and the effect of complexation with protein was analyzed in simple model systems by measuring photo-induced oxygen uptake in the presence and absence of BSA. Before irradiation, A2E was incubated with BSA for 5 min. In control samples, containing BSA with D_2_O-micelles, blue light induced very slow oxygen consumption with the rate 0.4 µM/min (Fig. 2a, b). In samples containing A2E solubilized in D_2_O/Triton X-100 micelles (without BSA) the rate of oxygen photouptake was at least threefold higher, and addition of BSA further accelerated the observable rate of oxygen photoconsumption. When micellar system was used, oxygen photoconsumption in H_2_O, compared to D_2_O, was almost twofold slower and azide reduced the rate of oxygen consumption by almost a factor of five, suggesting that the observed oxidation process might involve singlet oxygen (Fig. 3a, b). Without Triton X-100, the rate of oxygen photouptake in samples containing 0.1 mM BSA, was 2.5 times higher than that in micellar systems (Fig. 4). It indicates that complexation of A2E by BSA facilitates oxidation of the protein. The lower rate of oxygen photoconsumption observed in micellar systems containing BSA/A2E, suggests that the detergent could disturb complexation of BSA with A2E. The fact that azide had almost no effect on oxygen photoconsumption in samples containing BSA-A2E complexes (without Triton X-100) may suggest no role or only a minor role of singlet oxygen in the photooxidation of BSA complexed with A2E. It is possible that other ROS are involved. Indeed, our data indicate that superoxide anion photogenerated by A2E was involved in BSA oxidation mediated by A2E (vide infra). Since oxygen photoconsumption was slightly faster when SOD was added to the suspension of BSA and A2E in D_2_O (Supplementary Fig. 5S), it suggests competition between the interaction of superoxide with protein and SOD-catalyzed dismutation. However, the role of singlet oxygen cannot unambiguously be ruled out. Thus, the very weak inhibition of oxygen photoconsumption in the presence of 10 mM azide (Fig. 4), even if the process was mediated by singlet oxygen, could be due to limited accessibility of the protein sites where the binding with A2E occurs, particularly for negatively charged azide ions. It is expected that these binding sites should be more accessible to water molecules, which may explain the effect of H_2_O/D_2_O exchange. The observed enhancement of oxygen photoconsumption after exchanging H_2_O for D_2_O is consistent with the involvement of singlet oxygen, taking into account that in D_2_O singlet oxygen lifetime is about 15-fold longer than in H_2_O [40, 41].

Although A2E is considered to be a poor singlet oxygen generator [42, 43], it was reported by Sparrow and collaborators that singlet oxygen, photogenerated by this bis-retinoid, contributed to its photoreactivity [44] and phototoxicity [45]. In a study by Pawlak et al. [24] photo-induced oxygen uptake mediated by A2E, measured in a suspension of liposomes, containing unsaturated lipids, was modulated inversely by azide and histidine, which are physical and chemical quenchers of singlet oxygen, respectively [36]. It suggests the involvement of singlet oxygen, consistent with results obtained in this study, when micellar system was used. On the other hand, in complexes with proteins, such as BSA, A2E may operate via alternative mechanism, in which other ROS are involved.

### Singlet Oxygen Measurements

**Fig. 5:**
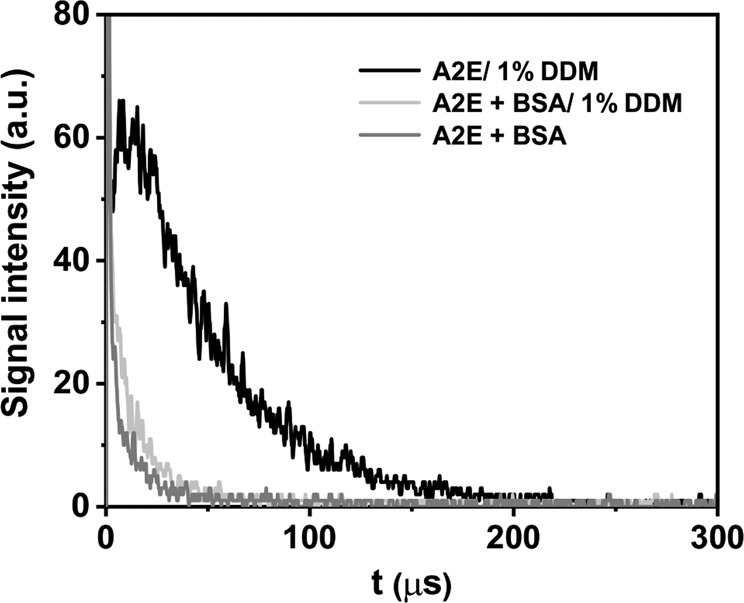
Time-resolved luminescence induced by photoexcitation of A2E in D_2_O: 1270 nm luminescence decay detected in A2E samples in D_2_O/DDM (black line) with addition of 100 µM BSA (light gray line) and with 100 µM BSA but without DDM (lower dark gray line). A2E was excited with 422 nm, 360 µJ laser pulses

Time-resolved measurements of phosphorescence in 1% DDM-D_2_O indicate that A2E is able to photogenerate singlet oxygen, with quantum yield estimated to be 1.9%. Micelles create specific microenvironment allowing the lipophilic A2E to dissolve. While aggregation of A2E, observed in water, reduces substantially the yield and lifetime of the chromophore excited triplet state, deaggregation of A2E increases its triplet state lifetime facilitating photogeneration of singlet oxygen. Due to higher solubility of oxygen in lipids, local concentration of dioxygen could be greater in micelles than in water, increasing the chance of A2E triplet state to form singlet oxygen. Singlet oxygen intrinsic lifetime is also longer in lipids/membranes in comparison to water (~15 µs compared to 3.5 µs in water). BSA greatly reduced the intensity of singlet oxygen phosphorescence and its lifetime (Fig. 5). The residual phosphorescence detected at 1270 nm, indicates that the yield of singlet oxygen photogenerated by the A2E complex with BSA is significantly lower than that by A2E in a micellar system. In addition, the lifetime of singlet oxygen photogenerated by BSA-A2E is reduced almost threefold, compared to the micellar system without BSA. This is due to efficient quenching by the protein, consistent with the reported corresponding rate constant being 5 × 10^8^ M^−1^s^−1^ [46].

### Generation of Free Radicals by Photoexcited A2E

**Fig. 6:**
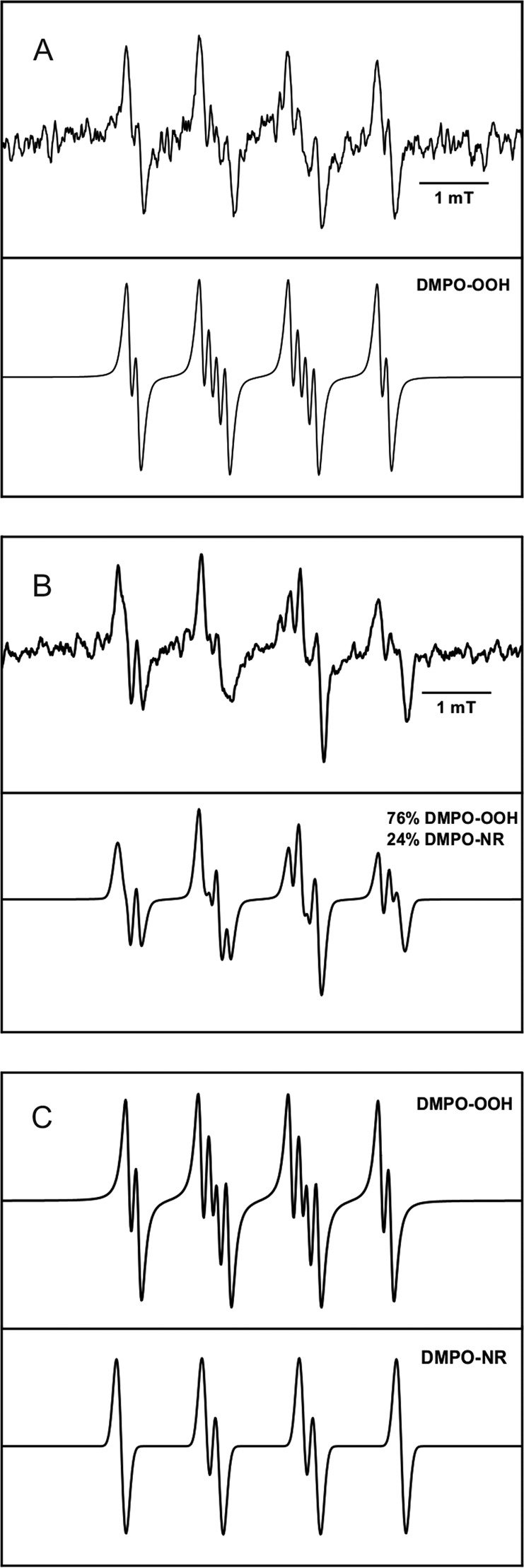
EPR spectra of detected (upper line) and simulated (lower line) DMPO adducts in the samples containing: **a** A2E in 80% DMSO after 30 min of blue-light irradiation. Hyperfine splitting constants were calculated as: *a*_N_ = 12.80 G, *a*_H_^β^ = 10.37 G, and *a*_H_^γ^ = 1.25 G; **b** A2E and BSA in 80% DMSO after 30 min of blue-light irradiation. **c** Simulations of individual DMPO spin adducts found in the A2E and BSA sample. Hyperfine splitting constants were calculated as: *a*_N1_ = 12.95 G, *a*_H1_^β^ = 10.44 G, *a*_H_^γ^ = 1.44 G, *a*_N2_ = 14.01 G, and *a*_H2_^β^ = 12.28 G

**Fig. 7:**
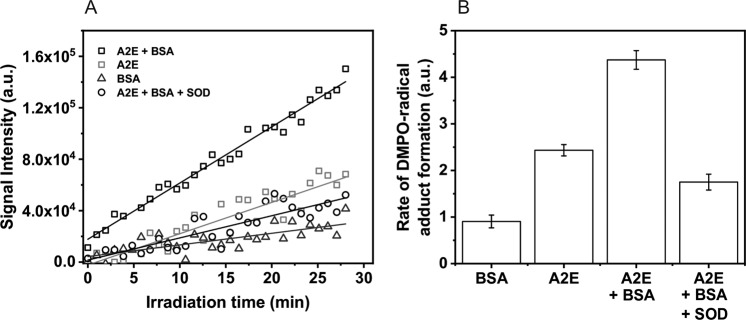
Kinetics (**a**) and accumulation rates (**b**) of DMPO-radical adducts formation in the samples containing A2E or BSA or A2E and BSA in 80% DMSO upon blue-light irradiation. Sample containing A2E and BSA was also blue light-irradiated with 500 units/mL of superoxide dismutase

Irradiation of A2E in a mixture of DMSO and water, in the presence of the DMPO spin trap, with blue light-induced generation of a spin adduct with hyperfine splitting consistent with trapping of superoxide anion [47]. It is shown in Fig. 6a, which compares the experimental and simulated EPR spectra. When BSA was added to A2E, the detected EPR signal (Fig. 6b) exhibited additional spectral features suggesting the formation of more than one spin adduct. Simulation of the observed spectrum, assuming the presence of DMPO adduct with superoxide and an unidentified spin adduct with hyperfine splitting typical for a nitrogen-centered radical [48] (Fig. 6c), indicates an acceptable fit of the experimental and simulated spectra. The kinetics of the DMPO spin adduct accumulation during sample irradiation indicates that the radical formation rate is enhanced by a factor of 1.8 when BSA was added (Fig. 7a, b). In addition, SOD reduced the rate of DMPO-OOH accumulation by a factor of 2.5.

Previous studies using EPR-spin trapping [49, 50] and pulse radiolysis [51] also indicated that A2E could generate superoxide anion in simple model systems. Pawlak et al. [24] demonstrated that A2E photogenerated superoxide radical four times faster than its photoreactive precursor, all-*trans* retinal. Nevertheless, the efficiency of superoxide anion photoproduction by A2E was determined to be very low—0.0003. Those data are consistent with the results obtained in this study. Spectral parameters of the detected spin adduct and the inhibitory effect of superoxide anion dismutase (SOD) on evolution of the spin adduct indicate that the main free radical photogenerated by A2E was superoxide anion. Interestingly, our data also showed that photogeneration of radicals mediated by A2E was enhanced by complexation of this bis-retinoid with BSA. A possible mechanism for the photoformation of superoxide anion by A2E could involve the following reactions:

$${\mathrm{A}}2{\mathrm{E}}\, +\,{\mathrm{h}}\nu\,\to\,^1{\mathrm{A}}2{\mathrm{E}}\,\to\,^3{\mathrm{A}}2{\mathrm{E}};\,^3{\mathrm{A}}2{\mathrm{E}}\, +\,{\mathrm{A}}2{\mathrm{E}}\,\to\,{\mathrm{A}}2{\mathrm{E}}^{ + \cdot }\,\\ +\,{\mathrm{A}}2{\mathrm{E}};{\mathrm{A}}2{\mathrm{E}}^{ - \cdot }\,+\,{\mathrm{O}}_2\,\to\,{\mathrm{A}}2{\mathrm{E}}\,+\,{\mathrm{O}}_2^{ - \cdot }.$$

In this scheme, self-quenching of the excited triplet state of A2E is responsible for the formation of its radical forms. Such processes have been observed for other photosensitizers, including rose Bengal [52]. The reactivity of the semireduced (A2E^−•^) and semioxidized (A2E^+•^) bis-retinoid was previously studied by pulse radiolysis [51]. That study clearly showed that the anion radical was relatively long-lived and interacted efficiently with dioxygen (the corresponding rate constant was determined to be 3 × 10^8^ M^−1^ s^−1^) forming superoxide anion, while the cation radical was short-lived and decayed rapidly. Of course, in the presence of an appropriate electron donor, the efficiency of the formation of the anion radical of A2E is expected to increase. BSA could serve as such an electron donor, as suggested by results of the spin trapping in samples containing BSA/A2E. To rule out possible role of singlet oxygen in the formation of the detected spin adducts, the spin trapping experiments were also carried out after addition of efficient quenchers of singlet oxygen—azide or DABCO. The corresponding results are shown in Supplementary material (Fig. 4S). It is apparent that azide not only increases the formation of the detected spin adduct by a factor of two (Fig. 4S C), but also modifies its spectral parameters (Fig. 4S A, B). As evident by simulation of the observed spectra, additional spin adduct is formed in the presence of azide (Fig. 4S A, B). Its spectral parameters are consistent with DMPO-N_3_ [53]. The observed data can be explained by assuming that azide acts as an electron donor for the excited triplet state of A2E:

$$^3{\mathrm{A2E}}\,+\,{\mathrm{N}}_3^ -\,\to\,{\mathrm{A2E}}^{ - \cdot }\,+\,{\mathrm{N}}_3^ \cdot ;{\mathrm{N}}_3^ \cdot\,+\,{\mathrm{DMPO}}\,\to\,{\mathrm{DMPO}}\,-\,{\mathrm{N}}_3.$$

Similar processes have been demonstrated in case of methylene blue used as a photosensitizer [54]. Importantly, when DABCO was added, no significant changes of the spin adducts were observed (no data shown). Our data clearly suggest that photo-induced electron transfer reactions (Type I photochemistry) determine the observed formation of superoxide anion and other radicals. It can be postulated that in the presence of azide, the azide radical is formed via electron transfer from the azide anion to the excited triplet state of A2E. The generated A2E anion radical via interaction with dioxygen forms additional superoxide anion (Fig. 4S C), while azide radical decays via unidentified reactions.

### Photooxidation of Albumin Mediated by A2E in Model Systems

**Fig. 8:**
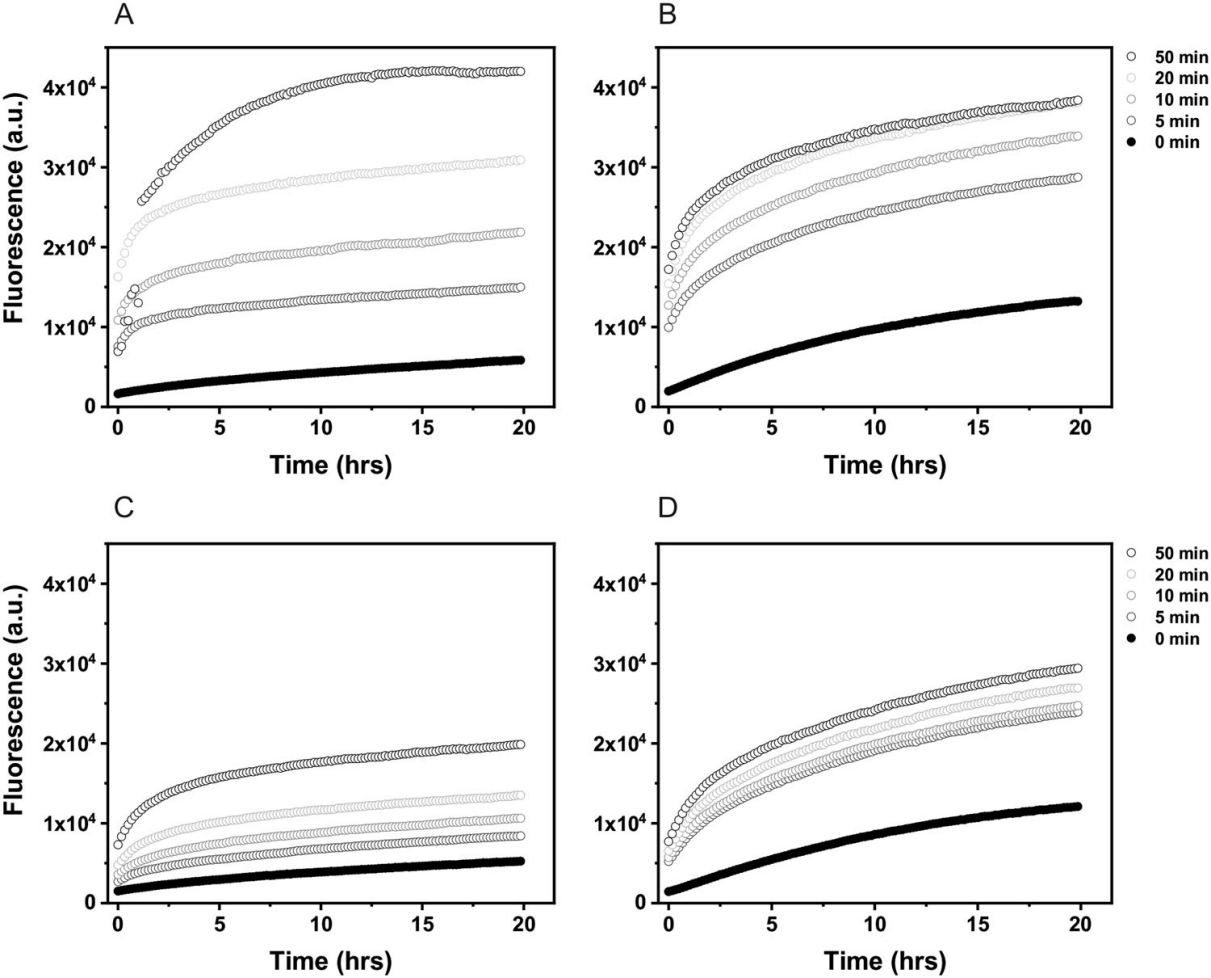
Evolution of the COH fluorescence in the samples containing 50 μM A2E and 100 μM BSA in D_2_O-micellar model system (**a**) or D_2_O-non-micellar model system (**b**) and H_2_O-micellar model system (**c**) or H_2_O-non-micellar model system (**d**) after irradiation with 440 nm light for selected time intervals

**Fig. 9:**
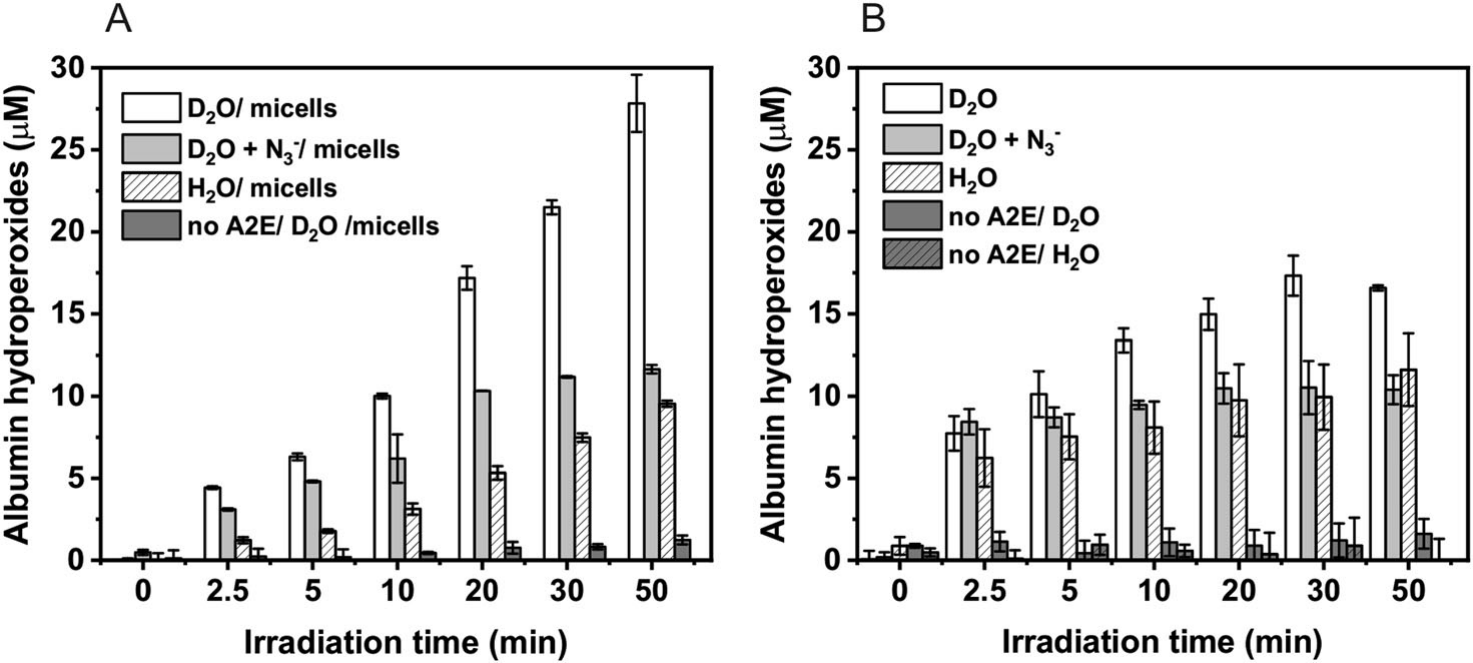
Concentration of albumin hydroperoxides generated in samples containing 50 μM A2E and 100 μM BSA in micellar model systems (**a**) and non micellar model systems (**b**) after irradiation with 440 nm light for selected time intervals

**Fig. 10:**
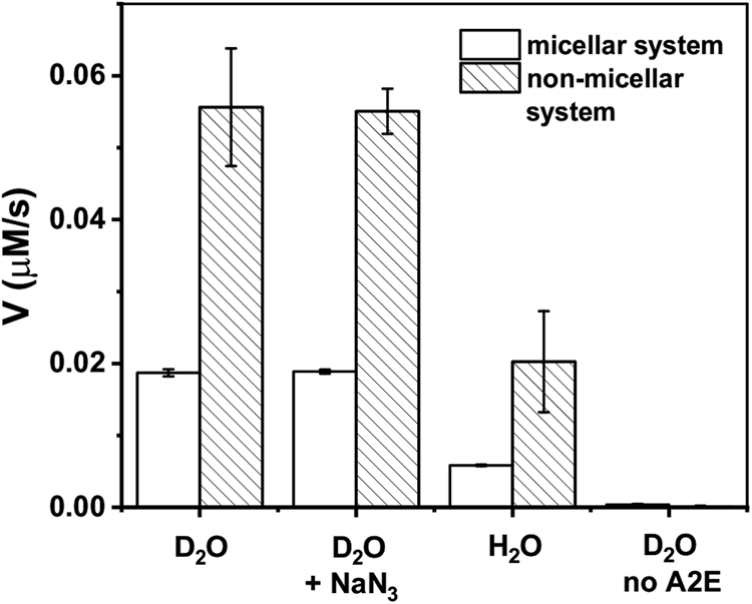
The initial formation of the albumin hydroperoxides (V) generated in the samples containing 50 μM A2E and 100 μM BSA in micellar model systems and non-micellar model systems after irradiation with 440 nm light

Photoreactivity of A2E was also examined as its ability to oxidize proteins. A simple model system consisting of BSA and A2E in H_2_O or D_2_O micellar and non-micellar systems was employed. Addition of the fluorogenic probe CBA to samples containing BSA and A2E, right after irradiation of the samples with blue light, resulted in a slow formation of the highly fluorescent hydroxycoumarin (COH)—product of the interaction of protein hydroperoxides with CBA [37, 38]. The kinetics of the COH accumulation suggests that the initial protein hydroperoxide formation is faster in D_2_O than in H_2_O, particularly in non-micellar model systems (Figs. 8, 9). Although addition of sodium azide did not inhibit the initial rate of COH formation, the amount of hydroxycoumarin accumulated after longer irradiation times was significantly lower than in the absence of azide (Fig. 10). This unexpected result could be explained assuming different penetration of BSA by azide ions at different state of the protein photosensitized oxidation. It cannot be ruled out that while the intact BSA is mostly impermeable for azide, oxidation of the protein by singlet oxygen and free radicals makes it more accessible for negatively charged ions such as azide.

Inhibition of protein hydroperoxides formation by sodium azide and slower oxidation of BSA in H_2_O compared to D_2_O confirm the involvement of singlet oxygen in the protein photooxidation mediated by A2E.

### Fluorescence Measurements

The interaction of A2E with BSA was studied by measuring the BSA fluorescence as a function of increasing concentration of A2E. To minimize aggregation of A2E in water and to ensure that the optical absorption of A2E and BSA during the titration experiment was acceptable for the analysis, concentrations of BSA and A2E were reduced 20-fold, in comparison with our other experiments. An exponential decay fitted to the obtained data (Supplementary Fig. 1S) demonstrated that dissociation constants for the A2E-BSA complex were comparable in solution and in the micellar system, with the corresponding values being *K*_d_ = 1.1 × 10^−5^ M and *K*_d_ = 9.2 × 10^−6^ M, respectively. Under the conditions used, binding of A2E to BSA was not disrupted by the presence of the detergent. The disrupting effect of the detergent was observed at higher concentrations of reagents (data not shown); however, at such higher concentrations, the OD of the sample was too high. A detail analysis of this issue requires further investigation by other techniques, such as differential scanning calorimetry.

**Fig. 11:**
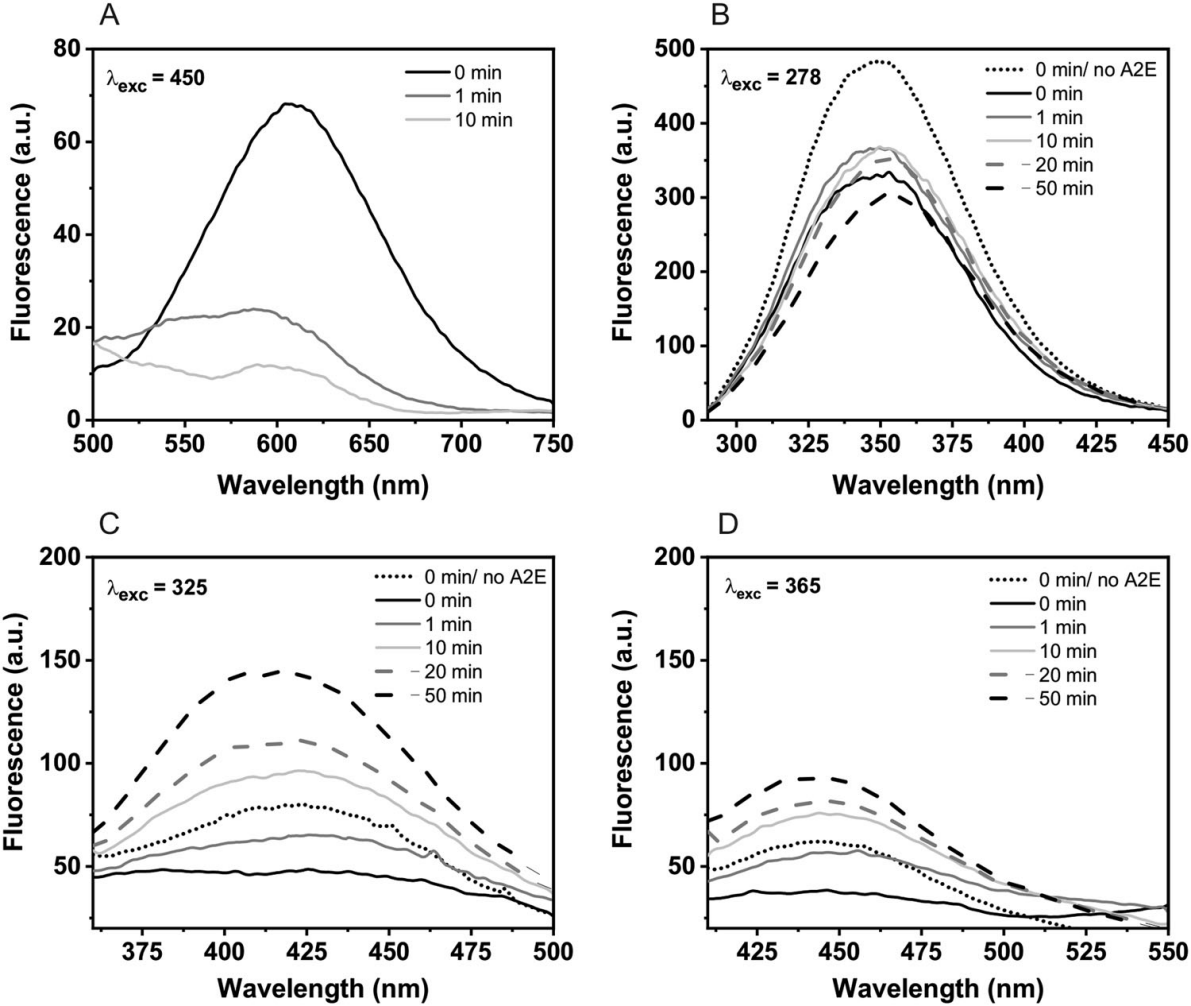
Fluorescence spectra of A2E (**a**), BSA (**b**), and tryptophan derivatives: N′-formylkynurenine (**c**) and kynurenine (**d**) in PB-D_2_O, recorded before (black lines) and after photooxidation mediated by A2E at selected time intervals. The excitation wavelengths are given in the top left-hand corners. Emission spectra of tryptophan and its derivatives before the addition of A2E to the BSA sample are marked as black dotted lines

Photooxidation of albumin mediated by A2E was monitored by measuring the protein fluorescence in D_2_O after excitation at 278, 325, and 365 nm, which corresponds to absorption maxima of tryptophan, N′-formylkynurenine, and kynurenine, respectively [39]. Kynurenines are oxidation products of Trp residues formed as a result of their interaction with singlet oxygen [55]. Data shown in Fig. 11 demonstrate that addition of A2E to solution of BSA caused quenching of the protein intrinsic fluorescence. Quenching of the tryptophan fluorescence may suggest that A2E binds to the protein site where Trp is located. After blue-light irradiation of the sample, which resulted in photobleaching of A2E, the characteristic emission of Trp at 350 nm moderately increased. Further irradiation of the sample with blue light brought about a decrease of the 350 nm emission, accompanied by an increase of the kynurenine emission in near visible region. Kynurenine is known to be a weaker fluorescence emitter and its fluorescence at 445 nm had lower intensity than fluorescence of N′-formylkynurenine at ~424 nm. Control experiments, in which BSA was irradiated with blue light, without A2E, showed no increase of the emission in the visible region (data not shown). These results confirmed that even after photobleaching of A2E, singlet oxygen was generated by excitation of its photodegradation products leading to further oxidation of BSA.

Additional experiments on A2E photodegradation have been performed using UV–Vis spectrophotometry. The absorption spectra recorded during irradiation of A2E in the presence of BSA in PB-D_2_O with blue light demonstrated that after 1 min irradiation almost all of A2E was photo-bleached (Supplementary Fig. 3S B). Absorption spectra of A2E in the micellar system (in the absence of BSA) showed that under such conditions this photoprocess is significantly slower. Thus, although photobleaching of A2E was also observed, even after irradiation of the sample for 10 min, the photobleaching of A2E was only partial (Supplementary Fig. 3S A). Photobleaching of A2E is accompanied by accumulation of its photodegradation products, which is apparent at 290 nm (Supplementary Fig. 3S A, C). This photodegradation product might be formed as a result of an internal double bond rearrangement in A2E molecules, which could be induced by electron transfer processes. It appears that photodegradation of A2E only partially depends on oxygen since it also occurred under conditions, in which oxygen content was greatly reduced by saturating the sample for 2.5 h with argon (Supplementary Fig. 3S C). Nevertheless, our data indicate that both oxygen and BSA accelerated the photodegradation and photooxidation of A2E.

### Circular Dichroism (CD)

As shown in Supplementary material (Fig. 2S), CD spectrum of BSA, exhibits a characteristic secondary structure with peaks of the α-helix (positive ellipticity at 192 nm) and a double-negative ellipticity (at 208 and 222 nm). In the presence of 0.8% acetonitrile, a slight reduction in negative ellipticity and an increase of the 192 nm band was observed. Following addition of A2E or irradiation of the protein in the presence of this bis-retinoid had no significant effect on the secondary structure of BSA. No changes in the recorded CD spectra of the secondary structure indicate that during the photooxidation of BSA mediated by A2E, no denaturation of the protein occurred.

## Summary and Conclusions

In conclusion, results of this study show that oxidizing photoreactivity of A2E is enhanced after complexation with BSA. Absorption spectra of A2E and changes in the protein intrinsic fluorescence, induced by titration with increasing concentration of A2E, demonstrate the formation of a noncovalent complexes of A2E with albumin (the corresponding dissociation constant was determined to be 1.1 × 10^−5^ M), which could be partially disturbed by the presence of a detergent. Significant quenching of the tryptophan fluorescence in BSA suggests that A2E may bind to the protein site where Trp residues are located. Quantum yield of singlet oxygen production by DDM-solubilized A2E in D_2_O is relatively low and was determined to equal 1.9%. Complexation of A2E by BSA significantly reduced the photogeneration of singlet oxygen by the bis-retinoid. On the other hand, the photoexcited A2E complexed by BSA generated free radicals with almost twofold higher efficiency than A2E in a homogeneous system. Superoxide anion was the main free radical generated by A2E-BSA. Simulation of the observed EPR spectra indicates that in the presence of BSA, an additional nitrogen-centered radical was photogenerated, which could result from BSA-mediated degradation of A2E. Aerobic photoexcitation of A2E-BSA complexes resulted in the formation of protein hydroperoxides, with the rate being faster in samples without the detergent. The inhibiting effect of sodium azide and the enhancement of H_2_O exchange for D_2_O suggest at least partial involvement of singlet oxygen in this process. This was confirmed by detecting characteristic fluorescence of the photoxidized BSA, consistent with the formation of N′-formylkynurenine and kynurenine, known singlet oxygen-dependent oxidation of tryptophan residues. Although A2E was photo-bleached during the first few minutes of blue-light irradiation, the photooxidation of BSA continued, suggesting that products of A2E photodegradation were also able to mediate the photooxidation process. The results from CD measurements indicated that the secondary structures of BSA practically did not change after photooxidation mediated by A2E, suggesting that no detectable denaturation of the protein occurred.

The ability of A2E to oxidize model protein upon excitation with blue light suggests that the pyridinium bis-retinoid may also oxidatively modify cellular proteins and change their key properties. Irreversible oxidative modifications, caused by retinoids, accumulated with age may have profound effect on the morphology and functions of retinal cells. Such alterations, occurring in the case of protein constituents of the RPE cytoskeleton, could have significant impact on nanomechanical properties of the cells, which are important for proper function of the human photoreceptor/RPE complex [56]. Although chronic oxidative stress may disturb many biological functions of RPE/photoreceptor complex, the key molecular targets that are subjected to oxidative modifications remain mostly unknown. We consider changes in the cytoskeleton of human RPE cells as one of the most sensitive indicators of oxidative modifications, which accompany chronic phototoxicity [29, 57]. Future research should focus on identification of the cytoskeletal proteins that might be oxidatively modified under chronic oxidative stress conditions, leading to disruption of key functions of the RPE/photoreceptor complex.

## Supplementary information

Figure 1S

Figure 2S

Figure 3S

Figure 4S

Figure 5S

Supplementary Data
