# Supplementary figures and images for: Photoreactivity of Bis-retinoid A2E Complexed with a Model Protein in Selected Model Systems
Source: Cell Biochem Biophys. 2020 Sep 12;78(4):415–27. doi: 10.1007/s12013-020-00942-1 (PMC7567710; doi:10.1007/s12013-020-00942-1)

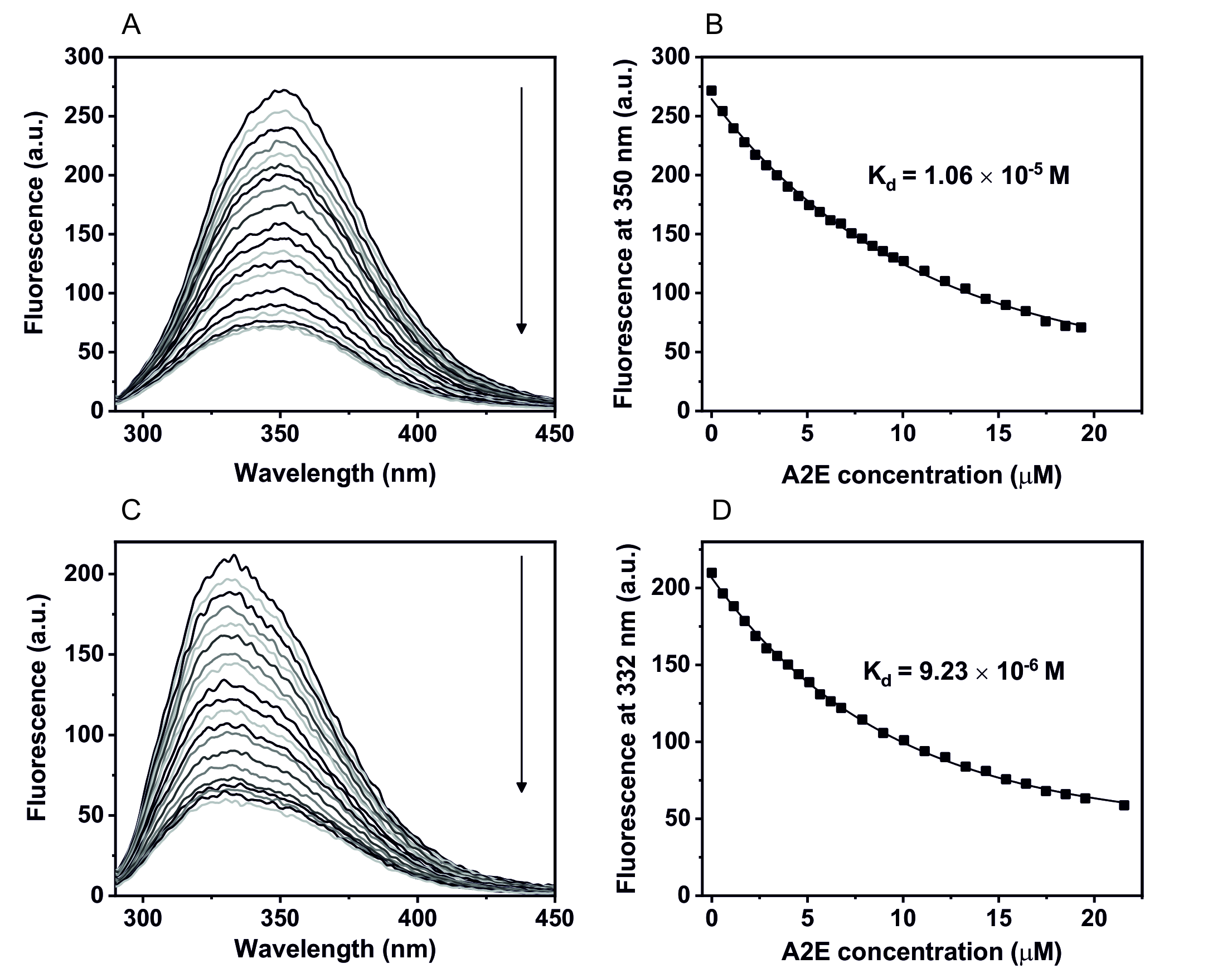

Supplement: Supplementary file 1 — Figure 1S [file 12013_2020_942_MOESM1_ESM.tif]

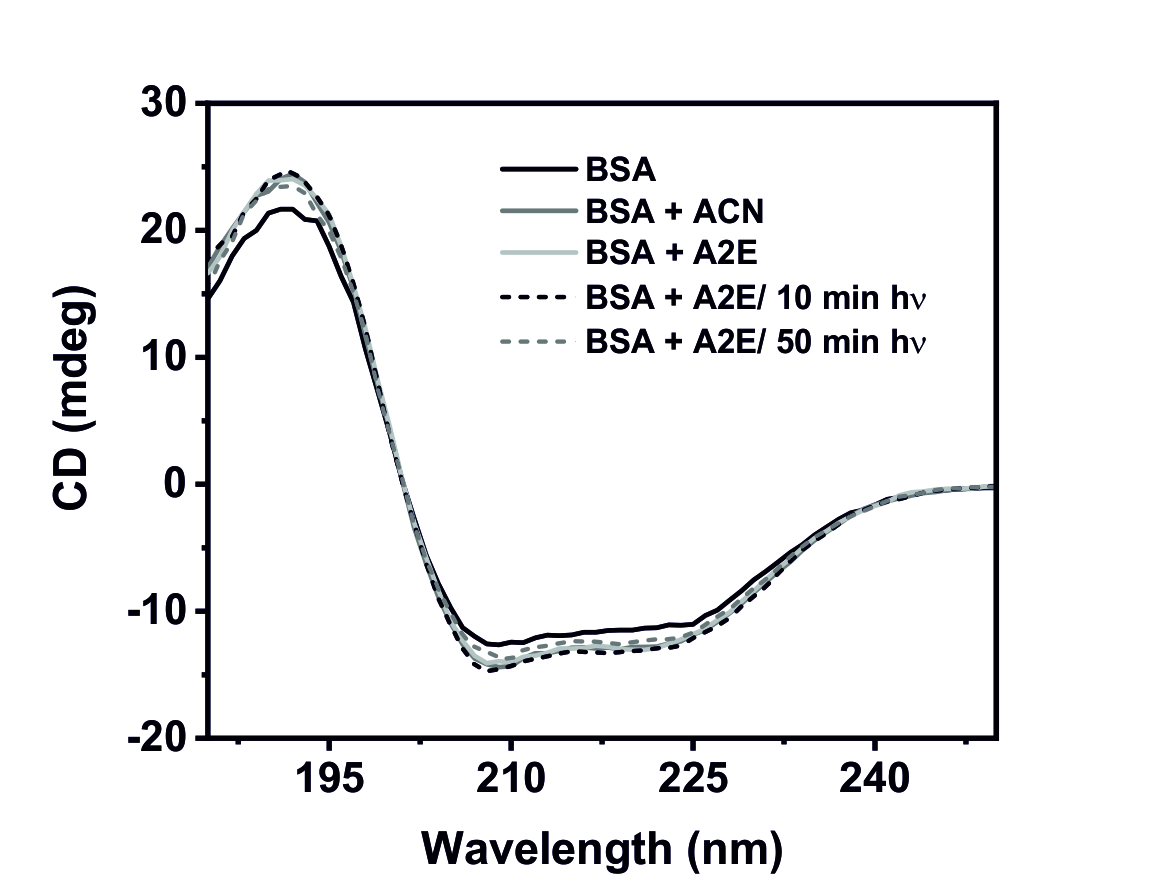

Supplement: Supplementary file 2 — Figure 2S [file 12013_2020_942_MOESM2_ESM.tif]

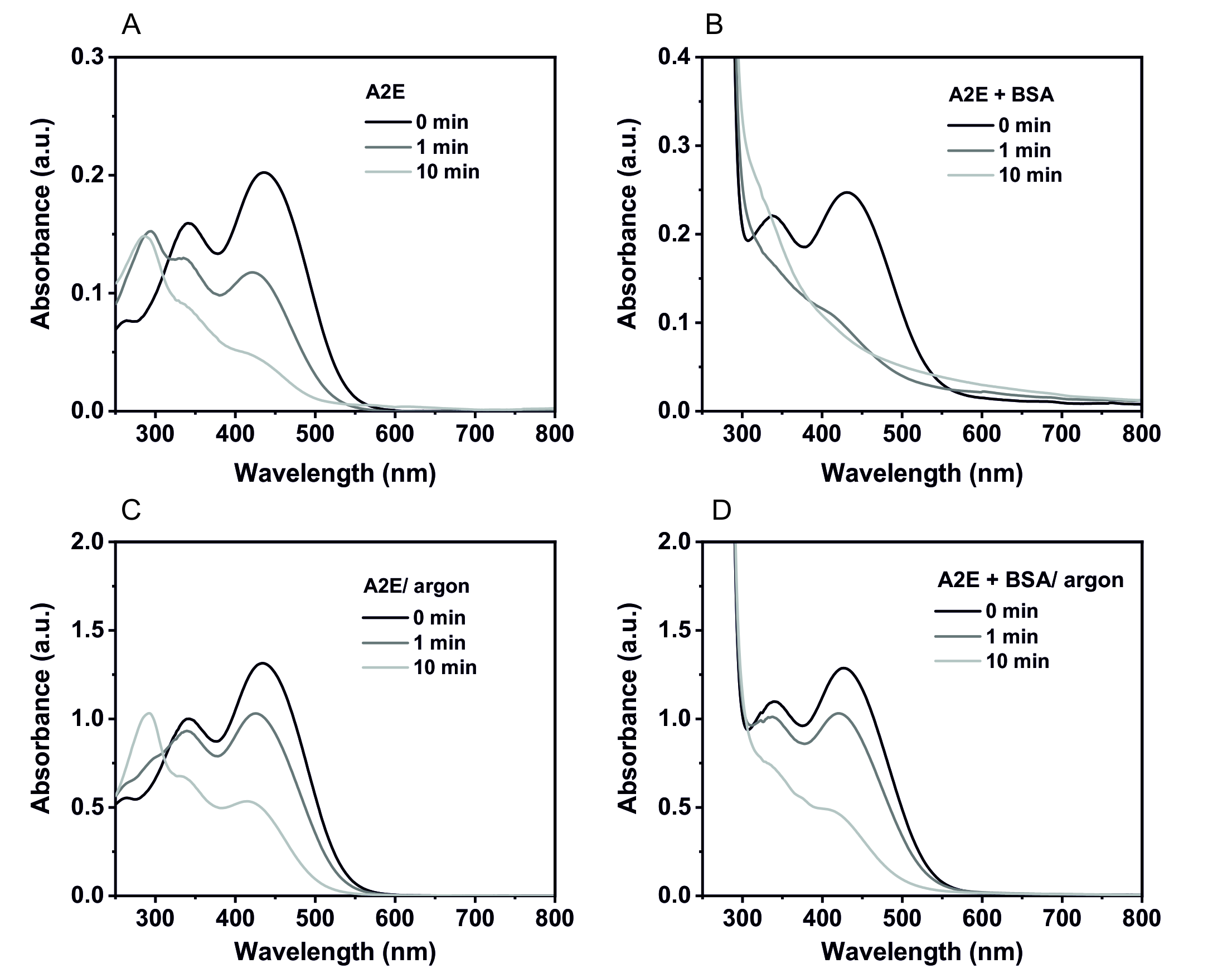

Supplement: Supplementary file 3 — Figure 3S [file 12013_2020_942_MOESM3_ESM.tif]

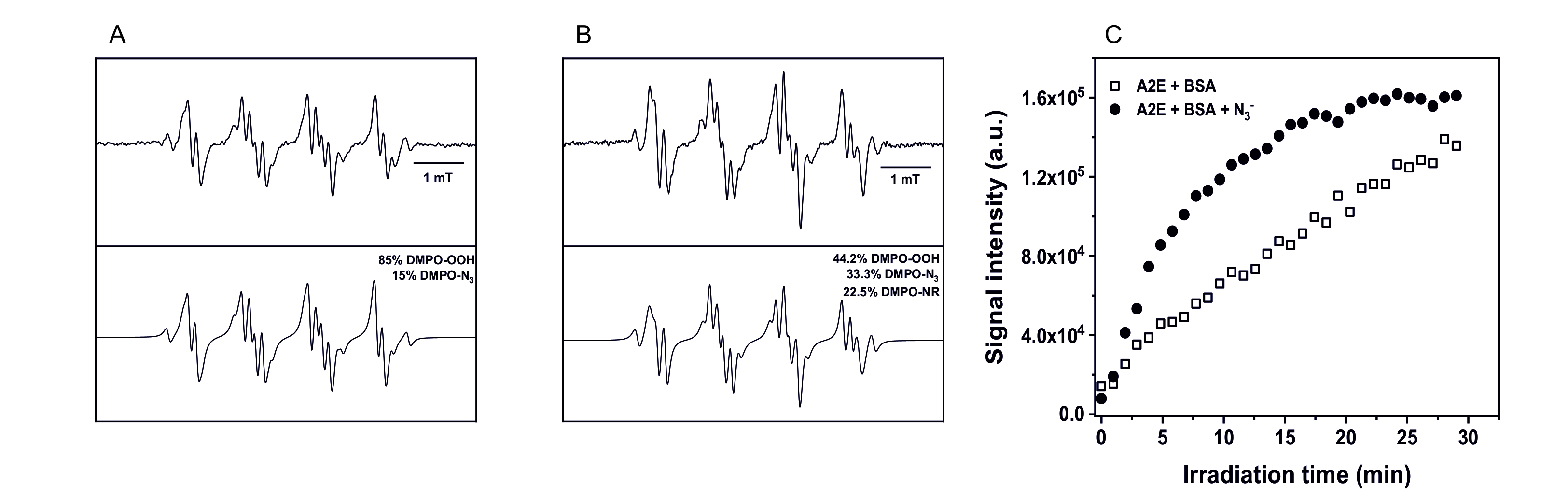

Supplement: Supplementary file 4 — Figure 4S [file 12013_2020_942_MOESM4_ESM.tif]

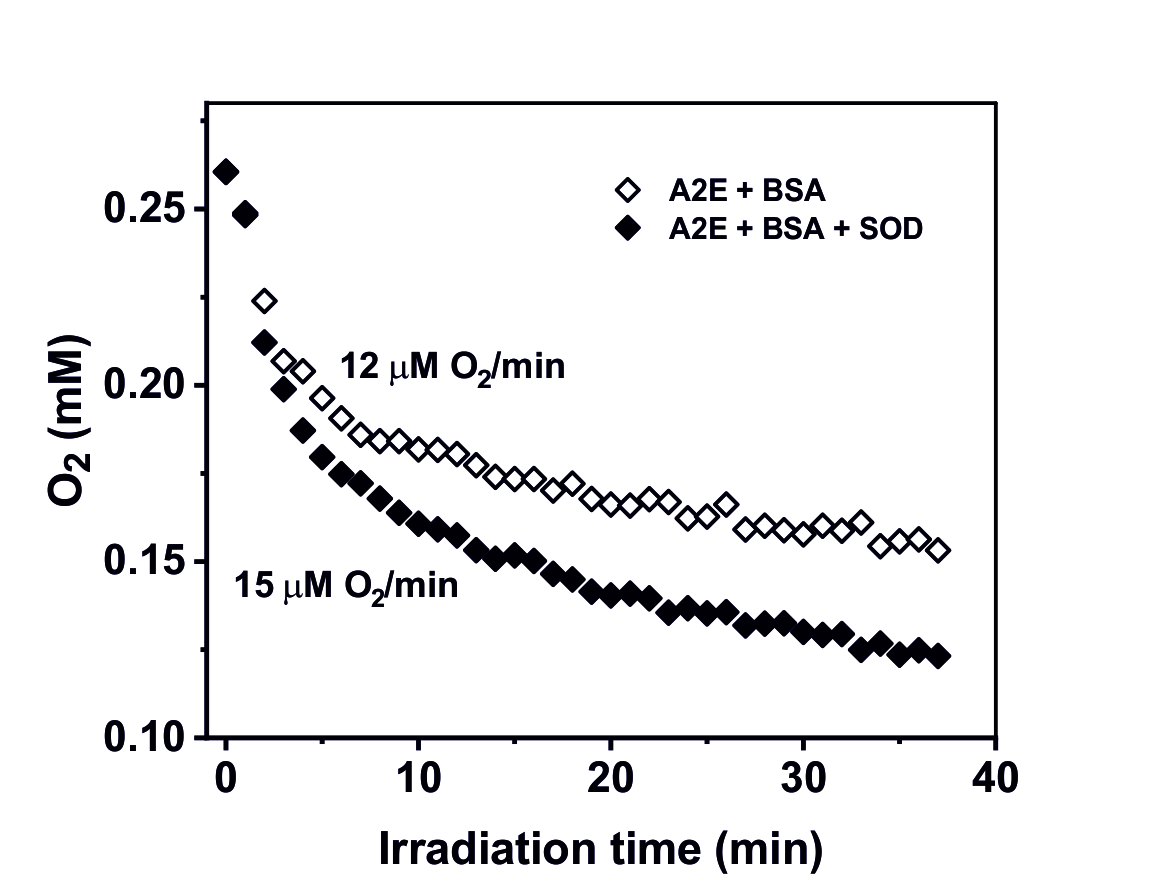

Supplement: Supplementary file 5 — Figure 5S [file 12013_2020_942_MOESM5_ESM.tif]
