# Supplementary material for: Photoreactivity of Bis-retinoid A2E Complexed with a Model Protein in Selected Model Systems
Source: Cell Biochem Biophys. 2020 Sep 12;78(4):415–27. doi: 10.1007/s12013-020-00942-1 (PMC7567710; doi:10.1007/s12013-020-00942-1)
Supplement: Supplementary file 6 — Supplementary Data [file 12013_2020_942_MOESM6_ESM.docx]

**Supplementary Data**

**Figure 1S.** Quenching of the BSA fluorescence with increasing A2E concentration. (A) BSA emission spectra recorded in PB-H_2_O after excitation at 278 nm and (B) dissociation constant (K_d_) of BSA-A2E complex in the solution determined using nonlinear regression fitted to the fluorescence at 350 nm plotted against A2E concentration. (C) BSA emission spectra recorded in PB-H_2_O in the presence of detergent (0.25% DDM) after excitation at 278 nm and (D) dissociation constant (K_d_) of BSA-A2E complex in the micellar system obtained using nonlinear regression fitted to the fluorescence at 332 nm plotted against A2E concentration. Arrows show the fluorescence change direction.

**Figure 2S.** CD spectra of: BSA (black solid line) recorded in PB-D_2_O; BSA after addition of acetonitrile (dark grey solid line); BSA after addition of A2E (grey solid line), and photooxidized BSA after 10 (black dashed line) and 50 min (grey dashed line) of blue light-irradiation in the presence of A2E.

**Figure 3S.** Absorption spectra of A2E recorded before (black line) and after 1 min (dark grey line) or 10 min (grey line) of blue light-irradiation under different conditions: (A) A2E in the D_2_O-micellar system; (B) A2E in the presence of BSA in D_2_O; (C) A2E in the D_2_O-micellar system after saturating the sample for 2.5 hrs with argon; (D) A2E in the presence of BSA in D_2_O after saturating the sample for 2.5 hrs with argon.

**Figure 4S.** EPR spectra of detected (upper line) and simulated (lower line) DMPO adducts in the samples containing: (A) A2E and NaN_3_ in 80% DMSO after 30 min of blue light-irradiation. Hyperfine splitting constants were calculated as: a_N1_ = 13.12 G, a_H1_^β^ = 10.61 G, a_H_^γ^ = 1.41 G (DMPO-OOH); a_N2_ = 13.94 G, a_H2_^β^ = 12.96 G, a_N_^β^ = 3.19 (DMPO-N_3_). (B) A2E, BSA and NaN_3_ in 80% DMSO after 30 min of blue light-irradiation. Hyperfine splitting constants were calculated as: a_N1_ = 13.04 G, a_H1_^β^ = 10.55 G, a_H_^γ^ = 1.57 G (DMPO-OOH); a_N2_ = 13.20 G, a_H2_^β^ = 14.79 G, a_N_^β^ = 2.64 (DMPO-N_3_); and a_N3_ = 13.92, a_H3_^β^ =12.28 G (DMPO-NR). (C) Kinetics of DMPO-radical adducts formation in the samples containing A2E and BSA or A2E, BSA and sodium azide in 80% DMSO upon blue light-irradiation.

**Figure 5S.** Photoinduced oxygen uptake in the samples containing A2E and BSA or A2E, BSA and 500 U/mL of SOD in PB-D_2_O upon blue-light irradiation.
